# Safety and efficacy of favipiravir in COVID-19 patients with pneumonia. A randomized, double-blind, placebo-controlled study (FAVID)

**DOI:** 10.1186/s41479-023-00124-6

**Published:** 2024-02-25

**Authors:** Juan P. Horcajada, Rebeca Aldonza, Mónica Real, Silvia Castañeda-Espinosa, Elena Sendra, Joan Gomez-Junyent, Inmaculada López-Montesinos, Silvia Gómez-Zorrilla, Silvia Briansó, Montserrat Duran-Taberna, Andrés Fernández, Cristina Tarragó, Teresa Auguet-Quintillá, the COVID-MAR Research group, Maria Arenas-Miras, Itziar Arrieta‐Aldea, Esperanza Cañas-Ruano, Roberto Güerri‐Fernandez, Hernando Knobel, Maria Milagro Montero, Ivan Pelegrín, Francisca Sánchez‐Martínez, Luisa Sorlí, Judith Villar‐García, the COVID-HJ23 group, Ajla Alibalic, Javier Camaron, Anna Maria Febrer, Laia Bertran, Andrea Barrientos

**Affiliations:** 1 Department of Infectious Diseases, Hospital del Mar. IMIM, Passeig Marítim 25, 08003 Barcelona, Spain, Universitat Pompeu Fabra (UPF), C/ del Dr. Aiguader, 88, 08003 Barcelona, Spain; 2 CIBERINFEC, CIBER of Infectious Diseases, Instituto de Salud Carlos III, C/ de Melchor Fernández Almagro, 3, 28029 Madrid, Spain; 3 Clinical Development Lead, Ferrer, Av. Diagonal, 549, 5°, 08029 Barcelona, Spain; 4 Service of the Internal Medicine, Hospital Universitari de Tarragona Joan XXIII, C/ Dr. Mallafrè Guasch, 4, 43005 Tarragona, Spain; 5 Universitat Rovira i Virgili, IISPV, C/ de Sant Llorenç, 21, 43201 Reus, Tarragona, Spain; 6 Internal Medicine Service, Hospital Sant Pau i Santa Tecla, Rambla Vella, 14, 43003 Tarragona, Spain; 7 Advanced Biotherapeutics Director, Ferrer, Av. Diagonal, 549, 5°, 08029 Barcelona, Spain; 8 R&D Project Lead, Ferrer, Av. Diagonal, 549, 5°, 08029 Barcelona, Spain; 9 GEMMAIR research group Institut d’Investigació Sanitària Pere Virgili (IISPV), C/ Dr. Mallafrè Guasch, 4, 43005 Tarragona, Spain

**Keywords:** Favipiravir, COVID-19, Pneumonia, Randomized clinical trial

## Abstract

**Purpose:**

To design a randomized clinical trial to assess the efficacy and safety of favipiravir in patients with COVID-19 disease with pneumonia.

**Methods:**

A randomized, double blind, placebo-controlled clinical trial of favipiravir in patients with COVID-19 pneumonia was conducted in three Spanish sites. Randomization 1:1 to favipiravir or placebo (in both groups added to the Standard of Care) was performed to treat the patients with COVID-19 pneumonia. The primary endpoint was “time to clinical improvement,” measured as an improvement for ≥ two categories on a 7-point WHO ordinal scale in an up to 28 days' time frame.

**Results:**

Forty-four patients were randomized (23 in the favipiravir group and 21 in the placebo group). The median time to clinical improvement was not different between the favipiravir and the placebo arms (10 days for both groups) and none of the secondary endpoints showed significant differences between arms.

The proportion of adverse events (both serious and non-serious) was statistically different between the favipiravir group (68.29%) and the placebo group (31.7%) (*p* = 0.019), but there was insufficient statistical evidence to correlate the degree of severity of the events with the treatment group.

**Conclusions:**

Favipiravir administered for ten days to patients with COVID-19 and pneumonia did not improve outcomes compared with placebo. Although this is an underpowered negative study, efficacy results align with other randomized trials. However, in the present study, the non-serious adverse events were more frequent in the favipiravir group.

## Introduction

The new severe acute respiratory syndrome coronavirus 2 (SARS-CoV2) is a single-stranded RNA virus, a member of the Betacoronavirus genus together with SARS-CoV1 and MERS-CoV. Aerial transmission during a short time of infectiousness [1, 2] has allowed SARS-CoV-2 to drive the pandemic that started in Wuhan, China, in December 2019, whose evolution has been shaped in waves since then and has not entirely ceased yet. Infection with SARS-CoV-2 is associated with acute respiratory symptoms and pneumonia. As of November 2022, 640 million patients were reported worldwide, of which 6.61 million have died [3].

While most patients with asymptomatic, mild, or moderate COVID-19 disease may recover without treatment, the benefit of pharmacological treatment is reserved for patients with severe disease or those with risk factors for progression to severe disease. After hesitant early beginnings with low-quality preliminary data available, supporting treatments that ultimately were shown to be ineffective, such as hydroxychloroquine [4], a few agents such as glucocorticoids [5], baricitinib [6], interleukin-6 inhibitors [7], and remdesivir [8] have demonstrated clinical benefit by decreasing either time to clinical recovery or mortality in severe cases [9].

Favipiravir (T-705) is an antipurine acid analogue approved in China and Japan for treating novel or re-emerging influenza virus infection in patients for which other anti-influenza drugs are either ineffective or not sufficiently effective. Favipiravir selectively inhibits the RNA polymerase of the influenza virus [10]. Therefore, it could also be effective for treating RNA viruses other than the influenza virus. In fact, favipiravir has been reported to be effective against the Ebola virus, Arenaviridae, and Bunyaviridae [11–13]. In addition, favipiravir inhibits the replication of SARS-CoV-2 in Vero E6 cells [14]. Results from two early clinical trials supporting improved outcomes with favipiravir triggered the interest in further investigation. Indeed, when favipiravir was administered to 35 COVID-19 Chinese patients along with interferon, clinical benefit over lopinavir/ritonavir was shown with a shorter time to resolution of fever and faster improvement of lung images [15]. Another placebo-controlled clinical trial showed a significantly shorter time to clinical cure (3 vs. 5 days; *p*=0.03) [16]. Therefore, these early data supported the efficacy of favipiravir in COVID-19 patients.

While no established treatment for COVID-19 is available at the time of this clinical trial, and the outbreak was expected to continue, research on new antiviral agents for the treatment of COVID-19 was urgently needed. Supposing the therapeutic effect of favipiravir on COVID-19 can be verified in humans, it could contribute to improve the prognosis of these patients. For all the above reasons, we designed this randomized clinical trial to assess the efficacy and safety of favipiravir in patients with COVID-19 disease with pneumonia.

## Methods

### Study design and patients

The study was conducted as a randomized, double-blind, multicenter clinical trial to compare favipiravir (1800 mg of favipiravir orally administered two times a day at least 4 hours apart on Day 1, then 800 mg orally administered twice daily on Day 2, and after that for up to 9 days) plus existing standard of care with placebo plus existing standard of care in COVID-19 patients with pneumonia. Three sites in Spain conducted the study between November 2020 and October 2021. Eligible patients had to be tested positive for SARS-CoV-2 on RT-PCR (reverse transcription polymerase chain reaction) test from respiratory specimens and were randomized 1:1 sequentially, according to a randomization list, through a web-based electronic system. Randomization was balanced by site, with an allocation sequence based on a block size of six. Both, participants and study staff were masked to treatment allocation.

Initial protocol was amended to reduce the mandatory hospitalization from 10 to 5 days, from the fifth month after the start of the study. Due to the high demand on health services throughout the country during the pandemic and the work overload of the participating sites, it was decided to continue the clinical follow-up of patients at home when a relevant clinical improvement was appreciated at hospital.

Patients were enrolled from November 2020 to September 2021, the follow-up period was 28 days. Patients included were adults 18 to 85 years old, with new lung lesions on chest image (chest CT or X-Ray), a SpO2< 94%, and at least two of the following signs: fever of 37.5°C or higher, respiratory rate ≥ 24/min, or cough. A negative influenza-test result was required during the epidemic peaks of the influenza virus season. Key exclusion criteria were as follows: increased procalcitonin levels (1 ng/ml or higher), abnormal NT-pro BNP (Natriuretic peptide tests) levels (400 pg/mL or higher), patients receiving immunosuppressants, and those patients previously treated with remdesivir. Either previous or current treatment with corticosteroids or tocilizumab was allowed.

The definition of the endpoints is listed in Table 1. The primary endpoint was “time to clinical improvement,” measured as an improvement for ≥ two categories on a 7-point WHO ordinal scale in an up to 28 days time frame. Secondary endpoints were duration of fever, time to discharge, or to a National Early Warning Score (NEWS) < 3 (whichever occurred first, only for the patients whose NEWS scores were ≥ 3 points at any time of the study) [17], time until weaning from oxygen therapy, time until weaning from mechanical ventilation, time to hospital discharge; exploratory endpoints were time to negative SARS-CoV-2 PCR Test, and time to positive SARS-COV2 antibody IgG Test.

**Table 1:** Efficacy endpoints

**Primary**	**All times from randomization date**
Time to clinical improvement measured as an improvement for ≥ two categories on a 7-point ordinal scale in an up to 28 days time-frame	Time to clinical improvement measured as improvement for ≥ two categories on a 7-point ordinal WHO scale (Time frame: up to 28 days):
	1. Not hospitalized, no limitations on activities;
	2. Not hospitalized, limitation on activities;
	3. Hospitalized, not requiring supplemental oxygen;
	4. Hospitalized, requiring supplemental oxygen;
	5. Hospitalized, on non-invasive ventilation or high flow oxygen devices;
	6. Hospitalized, on invasive mechanical ventilation or extracorporeal membrane oxygenation (ECMO);
	7. Death.
**Secondary**	
Duration of fever	Axillary temperature had returned to 37.2ºC or lower and was maintained at 37.2ºC or lower for at least 3 days.
Time to discharge or to a NEWS < 3 (whichever occurred first)	Only for all the patients whose NEWS scores were ≥ 3 points (at any time of the study) was defined as the duration until the patient was discharged or his/her total NEWS scores were maintained for 24 h under 3 points.
Time until weaning from oxygen therapy	Time until the patients who had a punctuation above 3 on the WHO ordinal scale of the clinical status, reached the category: “3. Hospitalized but not requiring supplemental oxygen” or lower and maintained it for 24 h.
Time until weaning from mechanical ventilation was defined as the	Time until the patients who had a punctuation of 6 on the WHO ordinal scale of the clinical status, reached the category: “5. Hospitalized, on non-invasive ventilation or high flow oxygen devices” or lower and maintain it for 24 h.
The time to hospital discharge	Time until the day of hospital discharge.
**Exploratory**	
Time to negative SARS-CoV-2 PCR Test	Days up to negative SARS-CoV-2 PCR Test.
Time to positive SARS-COV-2 antibody IgG test	Days up to positive SARS-COV-2 antibody IgG test.

### Treatment

The investigational product was favipiravir tablets containing 200 mg for oral administration. Doses regimen consisted of 1800 mg of favipiravir orally administered two times a day at least 4 hours apart on Day 1, then 800 mg orally administered twice daily on Day 2, and after that for up to 9 days. If required, a suspension of the study drug was prepared by adding water warmed to 55°C, to facilitate the administration per nasogastric tube. Reference treatment was supplied as tablets containing a matching placebo, identical to the favipiravir tablets with the same dose regimen (number of tablets and administration). In both arms, favipiravir, and placebo, the treatment duration was ten days, and existing treatment for COVID-19 was administered according to the current clinical practice for each participating site, including tocilizumab and corticosteroids by the investigator’s clinical judgment. Concurrent treatment with another antiviral agent, such as remdesivir, was not allowed.

### Statistical analyses

Due to the pilot nature of the study, no formal sample size estimation was performed. A total of 100 patients were planned to be included in the study: 50 in the favipiravir arm and 50 in the placebo arm. No interim analysis was planned. Three different populations were defined: the primary efficacy analysis set in this study was the modified Intent-To-Treat (mITT), including those patients confirmed to be positive for SARS-COV-2 on the RT-PCR test that received the study drug at least once. The Per Protocol set included those patients who meet the selection criteria, have the measures of the primary endpoints and do not have major deviations from the protocol. The Safety Analysis Set included those patients who received at least one interventional study drug administration.

All time-to-event endpoints were calculated for all patients in the mITT set using a Mantel-Haenszel log-rank test with Kaplan-Meier curves, Hazard Ratio estimates, and right censoring in the absence of event by day 28. Both displacements (WHO and NEWs scores) were analysed using the Hodges–Lehmann method (interpreted as negative association if the 95% confidence interval of the median contains the zero value) and the proportional odds model with the odds ratio (OR) being interpreted as the change in the score. A Cox regression model, using just the age at the start of the study as a covariate, was fitted to analyse fever resolution.

An additional post-hoc analysis was carried out to assess the statistical significance of proportion, causality and severity of the adverse events and also for serious adverse events.

### Regulatory and Ethics

The study was conducted in compliance with the protocol, regulatory requirements, data protection laws, good clinical practice, and the ethical principles of the last version (Fortaleza, 2013) of the Declaration of Helsinki. The Spanish Regulatory Competent Authority and the Central Ethics Committee approved the study on 1^st^ and 31^st^ July 2020, respectively, and all patients provided their written consent before study entry. The study is registered in EudraCT with the number 2020-002753-22.

## Results

### Patients disposition

**Fig. 1:**
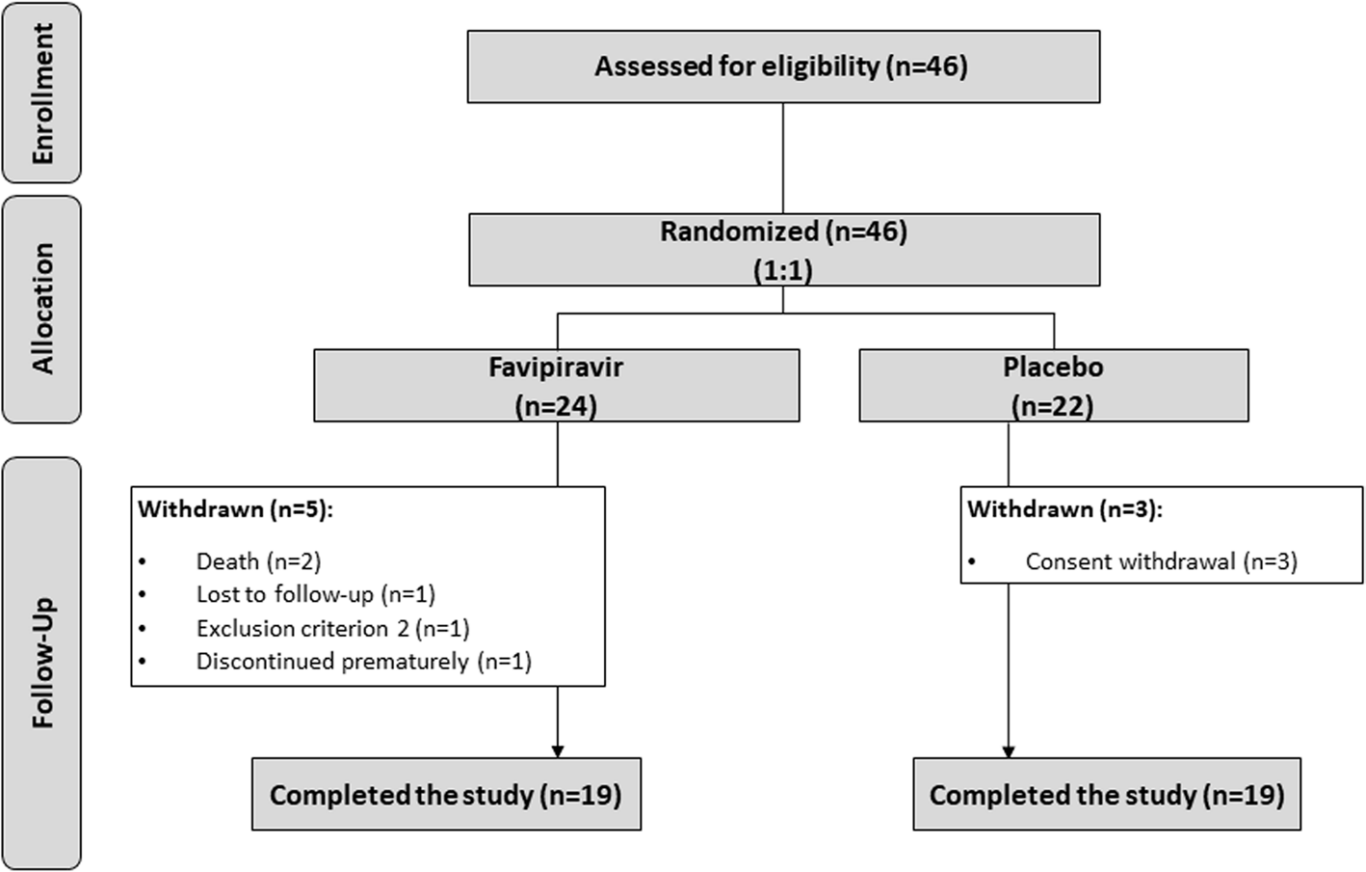
Flow-chart of the patients

A total of 46 patients were screened, and 44 were randomized (23 patients to the favipiravir group and 21 patients to the control group). Due to the wave-like behaviour of the pandemic, enrolment was exhausted and was therefore definitively discontinued without achieving the expected sample. One patient in the placebo group withdrew consent before receiving any doses, and one in the favipiravir group was excluded due to a NT-pro BNP above the allowed level. It was impossible to analyse her data for the mITT population as she exited the study without any follow-up data. Therefore, the mITT and the Safety Analysis Population included 44 patients (23 in the favipiravir group and 21 in the placebo group), while the Per Protocol population included 25 patients (13 in the favipiravir group and 12 in the placebo group). Figure 1 shows the trial’s flow chart.

### Baseline demographics

The median age of the patients was 52 years, with no significant differences between arms, and the majority were Caucasian (59%). There were more men (70.45%) than women (29.55%) in the study. The most common concurrent diseases were hypertension (16%), diabetes (9%), and cancer (9%), with no significant differences between arms. Regarding concomitant medications, all the patients had received corticosteroids, none had received antivirals, including remdesivir, or hydroxychloroquine, and one patient in each arm had received tocilizumab. Table 2 shows the baseline demographics and Table 3 the concomitant conditions of the patients.

**Table 2:** Baseline demographics

**Demographics**		**Favipiravir**	**Placebo**	**Total**
Gender, N (%)	Male	17 (73.91 %)	14 (66.67 %)	31 (70.45 %)
	Female	6 (26.09 %)	7 (33.33 %)	13 (29.55 %)
	Total	23 (100 %)	21 (100 %)	44 (100 %)
	Not available	0	0	0
Race, N (%)	Caucasian	12 (52.17 %)	14 (66.67 %)	26 (59.09 %)
	African	0 (0 %)	1 (4.76 %)	1 (2.27 %)
	Asian	3 (13.04 %)	3 (14.29 %)	6 (13.64 %)
	Other	8 (34.78 %)	3 (14.29 %)	11 (25 %)
	Total	23 (99.99 %)	21 (100.01 %)	44 (100 %)
	Not available	0	0	0
Age	N	23	21	44
	Mean (SD)	51.43 (11.43)	50.86 (9.92)	51.16 (10.61)
	Median (P25, P75)	51 (42.5, 60.5)	52 (45, 59)	51.5 (44.25, 59.75)
	Min, max	(33, 70)	(32, 64)	(32, 70)
	Not available	0	0	0
Height	N	23	21	44
	Mean (SD)	167.87 (10.85)	165.57 (9.12)	166.77 (10.01)
	Median (P25, P75)	168 (162, 172)	166 (163, 172)	167 (162, 172.5)
	Min, max	(146, 193)	(146, 179)	(146, 193)
	Not available	0	0	0
Weight	N	23	21	44
	Mean (SD)	79.94 (15.5)	77.94 (9.45)	78.99 (12.86)
	Median (P25, P75)	80 (72.75, 86.5)	75.8 (71.2, 81.8)	77.4 (71.42, 85)
	Min, max	(51, 120)	(64, 98)	(51, 120)
	Not available	0	0	0

Table for the mITT/SAS population

The denominator for the percentage is the number of subjects in each treatment arm

**Table 3:** Concomitant conditions

**Concomitant conditions**		**Favipiravir**	**Placebo**	**Total**
Hypertension N (%)	No	19 (82.61 %)	18 (85.71 %)	37 (84.09 %)
	Yes	4 (17.39 %)	3 (14.29 %)	7 (15.91 %)
	Total	23 (100 %)	21 (100 %)	44 (100 %)
	Not available	0	0	0
Diabetes N (%)	No	21 (91.3 %)	19 (90.48 %)	40 (90.91 %)
	Yes	2 (8.7 %)	2 (9.52 %)	4 (9.09 %)
	Total	23 (100 %)	21 (100 %)	44 (100 %)
	Not available	0	0	0
Chronic obstructive pulmonary disease (COPD) N (%)	No	23 (100 %)	20 (95.24 %)	43 (97.73 %)
	Yes	0 (0 %)	1 (4.76 %)	1 (2.27 %)
	Total	23 (100 %)	21 (100 %)	44 (100 %)
	Not available	0	0	0
Cardiovascular disease N (%)	No	23 (100 %)	20 (95.24 %)	43 (97.73 %)
	Yes	0 (0 %)	1 (4.76 %)	1 (2.27 %)
	Total	23 (100 %)	21 (100 %)	44 (100 %)
	Not available	0	0	0
Cancer N (%)	No	20 (86.96 %)	20 (95.24 %)	40 (90.91 %)
	Yes	3 (13.04 %)	1 (4.76 %)	4 (9.09 %)
	Total	23 (100 %)	21 (100 %)	44 (100 %)
	Not available	0	0	0
Autoimmune disease N (%)	No	23 (100 %)	21 (100 %)	44 (100 %)
	Yes	0 (0 %)	0 (0 %)	0 (0 %)
	Total	23 (100 %)	21 (100 %)	44 (100 %)
	Not available	0	0	0
Neurologic disease, N (%)	No	23 (100 %)	21 (100 %)	44 (100 %)
	Yes	0 (0 %)	0 (0 %)	0 (0 %)
	Total	23 (100 %)	21 (100 %)	44 (100 %)
	Not available	0	0	0
Other N (%)	No	20 (86.96 %)	15 (71.43 %)	35 (79.55 %)
	Yes	3 (13.04 %)	6 (28.57 %)	9 (20.45 %)
	Total	23 (100 %)	21 (100 %)	44 (100 %)
	Not available	0	0	0
Patients in whom concomitant conditions have affected the disease under study N (%)	No	17 (89.47 %)	18 (94.74 %)	35 (92.11 %)
	Yes	2 (10.53 %)	1 (5.26 %)	3 (7.89 %)
	Total	19 (100 %)	19 (100 %)	38 (100 %)
	Not available	4	2	6

Table for the mITT/SAS population

Percentages calculated using the total number of subjects in each treatment arm as the denominator

### Efficacy results

**Fig. 2:**
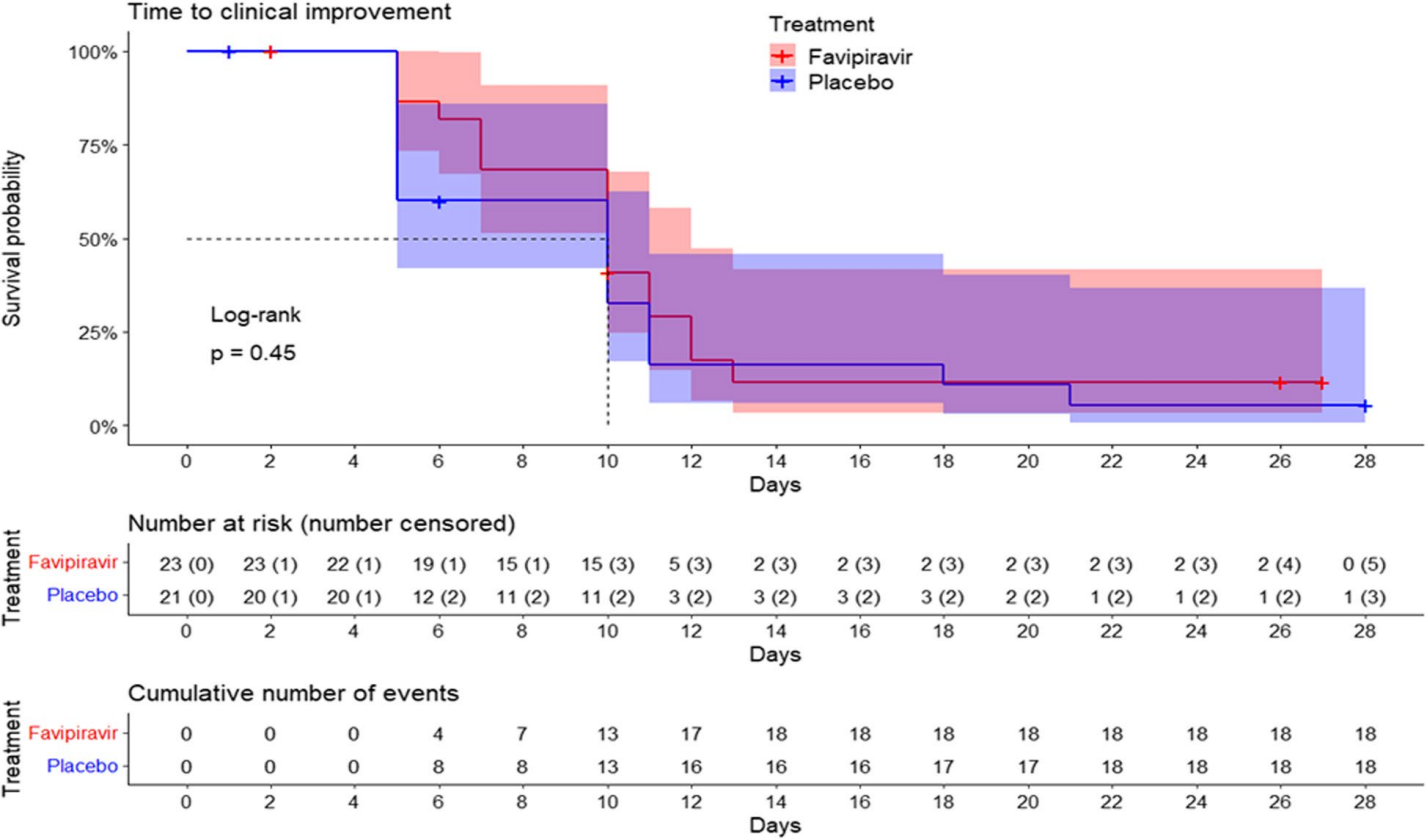
KM Curves. Time to clinical improvement by treatment group

The median time to clinical improvement in the mITT population, the primary endpoint of the study, was not different between the favipiravir and the placebo arms (10 days for both groups; *p*=0.45) (Fig. 2). Both median times to clinical improvement were also ten days in the Per Protocol population (*p*=0.83).

**Fig. 3:**
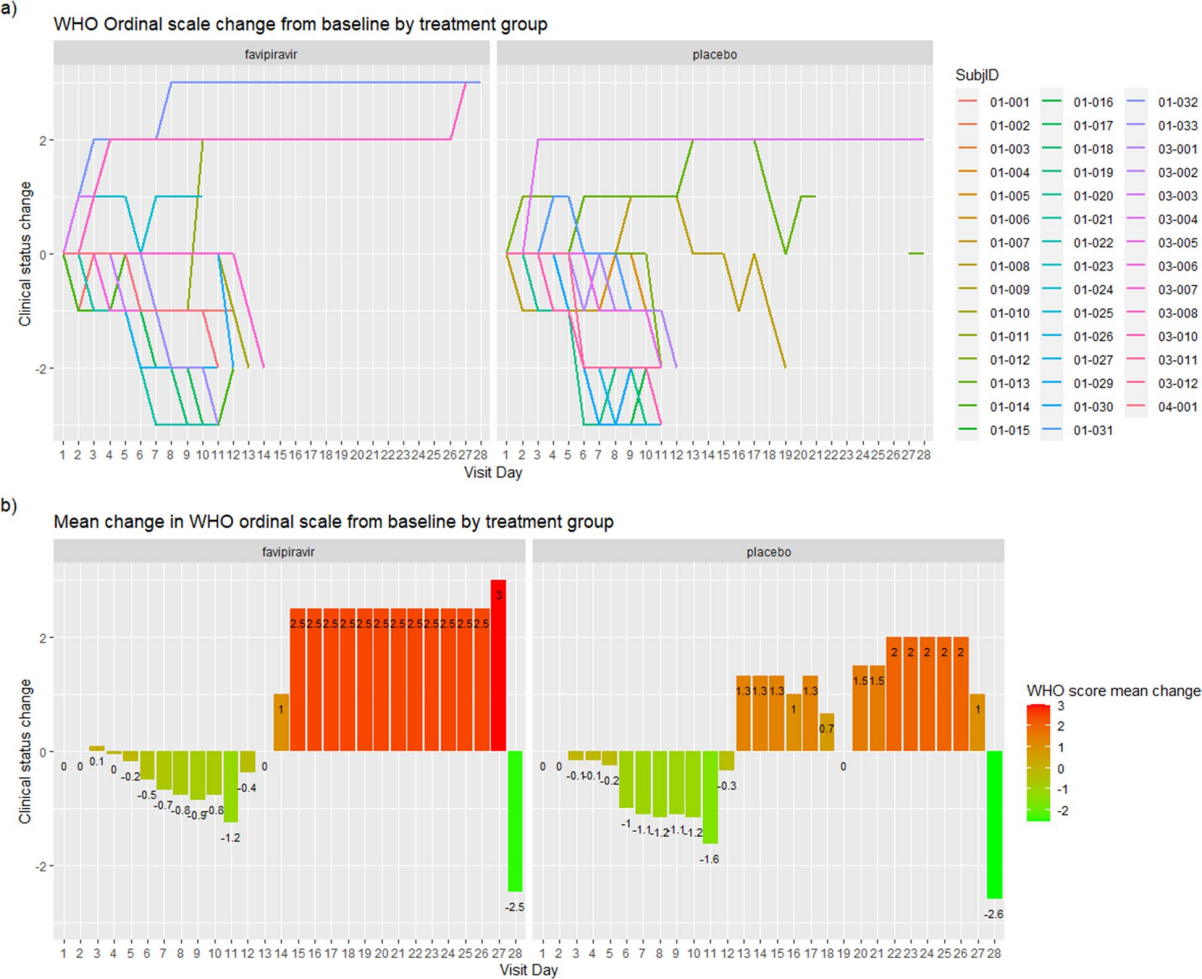
Change in WHO score from baseline

No differences were found between groups in any of the secondary efficacy analyses. The mean change in WHO ordinal scale displacement throughout the study by group (Fig. 3) showed similar results in both arms: a significant change over time with decreased values during the treatment period, followed by a dramatic increase and stabilization of the mean change in WHO score after treatment (OR=0.93[0.90–0.97]; *p*<0.001) with no significant association with treatment arm (OR=1.02[0.65–1.61]; *p*<0.923). This result was confirmed with The Hodges–Lehmann estimator [2.98 × 10−5 (−2.83 × 10^-6^, 2.49 × 10^-5^)].

The median duration of pyrexia was one day in both arms (*p*=0.93). Seventeen out of 44 patients (39%) in the mITT population were not included in the analysis on the time-to-fever resolution as they entered the trial with a temperature lower than 37.2ºC. Cox regression model, using the age at the start of the study as a covariate, was fitted to analyse fever resolution. According to the model, neither the treatment arm nor the patient’s age significantly changed the probability of recovering from fever (*p*=0.267 for arm and *p*=0.202 for age).

The median time to discharge or to NEWS < 3 for the favipiravir arm was five days, while for the placebo arm was six days (*p*=0.64). Significant displacement of the NEWS score was observed (OR=0.96 [0.93 – 0.99]; *p*=0.009) with no significant association with the treatment arm (OR=0.85 [0.54–1.35]; *p*=0.495). This result was confirmed with The Hodges–Lehmann estimator 2.04 × 10^-5^ (−3.05 × 10^-5^, 0.10). The median time to hospital discharge for both groups was ten days (*p*=0.56).

The median time to weaning from oxygen therapy for both groups was five days (*p*=0.64). Five patients needed mechanical ventilation (three in the favipiravir arm and two in the placebo arm).

No differences were found between groups in any of the exploratory efficacy analyses. Median time to negative SARS-CoV-2 PCR Test was 27 days and 12 days for the favipiravir and placebo arms, respectively (*p*=0.51), and medians of the time to positive SARS-CoV-2 Antibody Test were not in any of both groups reached (*p*=0.64).

### Safety results

Forty-four patients were included in the Safety Analysis Set (23 patients in the favipiravir arm and 21 in the placebo arm). A total of 41 adverse events (AE) were reported during the study: 29 non-Serious AE (non-SAEs) (20 in the favipiravir group and 9 in the placebo group) and 12 SAEs (8 in the favipiravir group and 4 in the placebo group).

The proportion of adverse events (both serious and non-serious) was statistically different between the favipiravir group (68.29%) and the placebo group (31.7%) (*p*=0.019), but there was insufficient statistical evidence to correlate the degree of severity/intensity of the events with the treatment groups (favipiravir and placebo) (*p*=0.658). The difference in the proportion of non-serious adverse events was statistically significant (*p*=0.041), with 68.97% and 31.03% adverse events in the study placebo and groups, respectively. However, there is insufficient statistical evidence to correlate causality (*p*=0.30) and severity (*p*=1) with the study group. Regarding Serious Adverse Events, no statistically significant differences were found between the two arms.

Gastrointestinal disorder was the System Organ Class (SOC) with the most reported non-SAEs: 18% of patients and 31% of all non-SAEs. Abdominal pain was the most frequent preferred term (PT) reported: 9.1% of patients and 13.8% of non-SAEs, whereas hypoxia was the most frequently reported SAE. Tables 4 and 5 lists AEs and SAEs experienced by the patients in any arm and is displayed by SOC and PT. Considering those adverse events that affect more than 5% of patients, psychiatric disorders (by SOC) and abdominal pain (by PT) were reported only in the favipiravir group (17.4% each).

**Table 4:** Cumulative incidence of Adverse Events (non-serious) in each treatment

**Adverse Events (non-serious)**	**Favipiravir Patients (*N*=23) N (%)**	**Favipiravir Events N (%)**	**Placebo Patients (*N*=21) N (%)**	**Placebo Events N (%)**	**Patients (*N*=44) N (%)**	**Events (*N*=29) N (%)**
**Total AEs**	**19 (82.61%)**	**20 (68.97%)**	**9 (42.86%)**	**9 (31.03%)**	**28 (63.64%)**	**29 (100%)**
**Gastrointestinal disorders**	5 (21.74 %)	6 (30 %)	3 (14.29 %)	3 (33.33 %)	8 (18.18 %)	9 (31.03 %)
Abdominal pain upper	4 (17.39 %)	4 (20 %)	0 (0 %)	0 (0 %)	4 (9.09 %)	4 (13.79 %)
Constipation	1 (4.35 %)	1 (5 %)	1 (4.76 %)	1 (11.11 %)	2 (4.55 %)	2 (6.9 %)
Abdominal pain	1 (4.35 %)	1 (5 %)	0 (0 %)	0 (0 %)	1 (2.27 %)	1 (3.45 %)
Diarrhea	0 (0 %)	0 (0 %)	1 (4.76 %)	1 (11.11 %)	1 (2.27 %)	1 (3.45 %)
Dyspepsia	0 (0 %)	0 (0 %)	1 (4.76 %)	1 (11.11 %)	1 (2.27 %)	1 (3.45 %)
**Infections and infestations**	3 (13.04 %)	3 (15 %)	2 (9.52 %)	2 (22.22 %)	5 (11.36 %)	5 (17.24 %)
Superinfection bacterial	2 (8.7 %)	2 (10 %)	0 (0 %)	0 (0 %)	2 (4.55 %)	2 (6.9 %)
Clostridium difficile infection	1 (4.35 %)	1 (5 %)	0 (0 %)	0 (0 %)	1 (2.27 %)	1 (3.45 %)
Hematoma infection	0 (0 %)	0 (0 %)	1 (4.76 %)	1 (11.11 %)	1 (2.27 %)	1 (3.45 %)
Urinary tract infection	0 (0 %)	0 (0 %)	1 (4.76 %)	1 (11.11 %)	1 (2.27 %)	1 (3.45 %)
**Psychiatric disorders**	4 (17.39 %)	4 (20 %)	0 (0 %)	0 (0 %)	4 (9.09 %)	4 (13.79 %)
Insomnia	2 (8.7 %)	2 (10 %)	0 (0 %)	0 (0 %)	2 (4.55 %)	2 (6.9 %)
Anxiety	1 (4.35 %)	1 (5 %)	0 (0 %)	0 (0 %)	1 (2.27 %)	1 (3.45 %)
Nightmare	1 (4.35 %)	1 (5 %)	0 (0 %)	0 (0 %)	1 (2.27 %)	1 (3.45 %)
**General disorders and administration site conditions**	1 (4.35 %)	1 (5 %)	1 (4.76 %)	1 (11.11 %)	2 (4.55 %)	2 (6.9 %)
Chest discomfort	1 (4.35 %)	1 (5 %)	0 (0 %)	0 (0 %)	1 (2.27 %)	1 (3.45 %)
Chest pain	0 (0 %)	0 (0 %)	1 (4.76 %)	1 (11.11 %)	1 (2.27 %)	1 (3.45 %)
**Nervous system disorders**	2 (8.7 %)	2 (10 %)	0 (0 %)	0 (0 %)	2 (4.55 %)	2 (6.9 %)
Cerebral venous thrombosis	1 (4.35 %)	1 (5 %)	0 (0 %)	0 (0 %)	1 (2.27 %)	1 (3.45 %)
Headache	1 (4.35 %)	1 (5 %)	0 (0 %)	0 (0 %)	1 (2.27 %)	1 (3.45 %)
**Vascular disorders**	1 (4.35 %)	1 (5 %)	1 (4.76 %)	1 (11.11 %)	2 (4.55 %)	2 (6.9 %)
Hypotension	1 (4.35 %)	1 (5 %)	0 (0 %)	0 (0 %)	1 (2.27 %)	1 (3.45 %)
Thrombophlebitis	0 (0 %)	0 (0 %)	1 (4.76 %)	1 (11.11 %)	1 (2.27 %)	1 (3.45 %)
**Ear and labyrinth disorders**	1 (4.35 %)	1 (5 %)	0 (0 %)	0 (0 %)	1 (2.27 %)	1 (3.45 %)
Tinnitus	1 (4.35 %)	1 (5 %)	0 (0 %)	0 (0 %)	1 (2.27 %)	1 (3.45 %)
**Investigations**	0 (0 %)	0 (0 %)	1 (4.76 %)	1 (11.11 %)	1 (2.27 %)	1 (3.45 %)
Hepatitis B virus test positive	0 (0 %)	0 (0 %)	1 (4.76 %)	1 (11.11 %)	1 (2.27 %)	1 (3.45 %)
**Metabolism and nutrition disorders**	1 (4.35 %)	1 (5 %)	0 (0 %)	0 (0 %)	1 (2.27 %)	1 (3.45 %)
Hypertriglyceridemia	1 (4.35 %)	1 (5 %)	0 (0 %)	0 (0 %)	1 (2.27 %)	1 (3.45 %)
**Respiratory, thoracic and mediastinal disorders**	0 (0 %)	0 (0 %)	1 (4.76 %)	1 (11.11 %)	1 (2.27 %)	1 (3.45 %)
Respiratory failure	0 (0 %)	0 (0 %)	1 (4.76 %)	1 (11.11 %)	1 (2.27 %)	1 (3.45 %)
**Skin and subcutaneous tissue disorders**	1 (4.35 %)	1 (5 %)	0 (0 %)	0 (0 %)	1 (2.27 %)	1 (3.45 %)
Pruritus	1 (4.35 %)	1 (5 %)	0 (0 %)	0 (0 %)	1 (2.27 %)	1 (3.45 %)

**Table 5:** Cumulative incidence of Serious Adverse Events in each treatment

**Serious Adverse Events**	**Favipiravir Patients (*N*=23) N (%)**	**Favipiravir Events N (%)**	**Placebo Patients (*N*=21) N (%)**	**Placebo Events N (%)**	**Patients (*N*=44) N (%)**	**Events (*N*=12) N (%)**
**Total SAEs**	**5 (21.74%)**	**8 (66.67%)**	**4 (19.05%)**	**4 (33.33%)**	**9 (20.45%)**	**12 (100%)**
**Respiratory, thoracic and mediastinal disorders**	3 (13.04 %)	5 (62.5 %)	3 (14.29 %)	3 (75 %)	6 (13.64 %)	8 (66.67 %)
Hypoxia	3 (13.04 %)	3 (37.5 %)	3 (14.29 %)	3 (75 %)	6 (13.64 %)	6 (50 %)
Pneumomediastinum	1 (4.35 %)	1 (12.5 %)	0 (0 %)	0 (0 %)	1 (2.27 %)	1 (8.33 %)
Pneumothorax	1 (4.35 %)	1 (12.5 %)	0 (0 %)	0 (0 %)	1 (2.27 %)	1 (8.33 %)
**Nervous system disorders**	1 (4.35 %)	2 (25 %)	1 (4.76 %)	1 (25 %)	2 (4.55 %)	3 (25 %)
Cerebral venous thrombosis	1 (4.35 %)	1 (12.5 %)	0 (0 %)	0 (0 %)	1 (2.27 %)	1 (8.33 %)
Hemorrhage intracranial	1 (4.35 %)	1 (12.5 %)	0 (0 %)	0 (0 %)	1 (2.27 %)	1 (8.33 %)
Seizure	0 (0 %)	0 (0 %)	1 (4.76 %)	1 (25 %)	1 (2.27 %)	1 (8.33 %)
**Renal and urinary disorders**	1 (4.35 %)	1 (12.5 %)	0 (0 %)	0 (0 %)	1 (2.27 %)	1 (8.33 %)
Renal failure	1 (4.35 %)	1 (12.5 %)	0 (0 %)	0 (0 %)	1 (2.27 %)	1 (8.33 %)

In the favipiravir arm, the most reported SOC were gastrointestinal disorders (22% of the patients and 30% of all non-SAEs) and psychiatric disorders (17% of patients and 20% of all non-SAEs). In the placebo group, the most reported SOC were gastrointestinal disorders (14.3% of patients and 33% of all non-SAEs) and infections and infestations (9.5% of patients and 22% of all non-SAEs).

Most non-SAEs were mild and moderate in severity, 76% and 17%, respectively. Only two non-SAEs (6.8%) were severe. Regarding causal relationships, most non-SAEs were considered unrelated (72%) or unlikely related (10.4%). Only 13.8% and 3.5% of non-SAEs were considered probably related and related, respectively. Lastly, there were only three grade 3 and 4 non-SAEs: *Clostridium difficile* infection, cerebral venous thrombosis, and respiratory failure. Two deaths in the favipiravir group were reported during the study, one due to intracranial haemorrhage, which was considered unrelated to investigational treatment, and the other by renal failure, which was assessed by the investigator as possibly associated with the investigational treatment.

## Discussion

The results of this study show that favipiravir did not result in improved outcomes (time to clinical improvement) compared with placebo when used to treat patients with COVID-19 pneumonia. Multiple secondary endpoints (duration of fever, time to discharge or a National Early Warning Score (NEWS) < 3, time until weaning from oxygen therapy, time until weaning from mechanical ventilation, time to hospital discharge) or exploratory endpoints (time to negative SARS-CoV-2 PCR Test, and time to positive SARS-COV-2 antibody IgG Test) did not show differences between groups as well, and additional sensitive analysis performed in the Per Protocol population confirmed the results observed in the mITT population. However, the study did not have enough statistical power to find any differences. In fact, this is an underpowered negative study because of exhaustion of enrolment. Exhaustion of patients’ enrolment over time has been observed in COVID-19 clinical trials. The temporal evolution of the pandemic has taken place in the form of successive waves. This has meant that the trials have had variable enrolment rates over time and that, at the end of the waves, many trials had exhausted their enrolment potential (sites), as has been the case in this study.

Our negative result aligns with previous randomized clinical trials comparing favipiravir with a placebo. Indeed, favipiravir has been shown not to improve outcomes compared with placebo regarding time to virological response [18], time to progression to hospitalization [18, 19], or time to progression to pneumonia [19, 20] when administered to treat early symptomatic COVID-19 infection. Nor has it shown any impact on time to clinical improvement in moderate symptomatic COVID-19 disease [21, 22]. Other trials comparing with a no-treatment arm [23] have also shown no benefit from favipiravir. In a recent trial, favipiravir did not improve clinical outcomes in all patients admitted to hospital with COVID-19. However, patients younger than 60 years seemed to have a beneficial clinical response [24]. Moreover, a meta-analysis including nine studies showed a significant clinical improvement in the favipiravir group versus the control group during seven days after hospitalization. However, supplemental oxygen therapy requiring, transfer to ICU, adverse events and mortality were similar in both groups. Authors concluded that favipiravir possibly exerted no significant beneficial effect in the term of mortality in the general group of patients with mild to moderate COVID‑19 [25].

The tolerability of favipiravir, when used to treat patients with COVID-19 disease, is considered predictable and manageable [24–26]. In our trial, more adverse effects were detected in the favipiravir group, but these were non-SAEs. In addition, we did not find sufficient statistical evidence to correlate the degree of severity/intensity of the events with the treatment group. Furthermore, laboratory values, vital signs, physical examination, and imaging tests did not reveal any major issues concerning the safety of favipiravir. Two patients died during the study. Both belonged to the favipiravir group. In one of them death was not considered drug related. In the other case, the cause of death was renal failure. The physicians considered that it could be related to the drug, since no other cause was found. However, in a recent study favipiravir seemed a suitable therapeutic option in pediatric patients affected by COVID-19 with kidney injury without a need for dose adjustment [27] and in another study appeared to be well tolerated in adults with renal failure [28].

This study has some strengths and some weaknesses. The endpoints depicted a variety of clinical parameters, including clinical severity and virologic parameters, which led to a comprehensive assessment of the patients. Also, the randomized design was a strength before the initiation of the study. This trial emphasizes the need for randomized controlled trials to show the highest level of evidence after the widespread use in the early times of the pandemic of treatments that have subsequently been shown to be inactive for treating COVID-19 disease. However, the small sample size, has ultimately been the most marked weakness of the study, leading to an underpowered study that precludes firm conclusions.

As a future prospect, several issues must be resolved. A particular problem is the post COVID-19 syndrome, which is troublesome for patients and may last for several weeks or months, leading to long symptomatic periods for the patients and persistent loss of work productivity. Post COVID-19 syndrome is related to an immune response with cytokine release [29] and prolonged pro-coagulant status [30], requiring multidisciplinary management [31] and representing a real challenge for the health system. On the other hand, transitioning from pandemic to endemic, COVID-19 disease is permanently among us. After social distance rules have been relaxed, vaccination programs must be potentiated. For all these reasons, investigation on antivirals is warranted. Whether favipiravir may be more active against COVID-19 when administered for more than ten days, which is the duration of favipiravir treatment in our study, or whether it could be more efficient when administered earlier during the course of the disease, or whether it is more active in patients of Asian ethnicity, or whether it is more effective in younger than 60 years people, are still unanswered questions. It is worth noting that 14 days of treatment has been commonly used in previous clinical trials that have shown a significant clinical improvement on day 14 compared to day 7, as reported in the meta-analysis results [26]. Of note, these results were reported in May 2021. The conclusions supported that favipiravir was a promising agent to treat COVID-19, based on evidence of viral clearance and the aforementioned clinical benefit on day 14. The same positive view was highlighted in a review published in January 2021 (Joshi 2021). The history of favipiravir to treat COVID-19 has evolved from an initial enthusiasm [26, 32] to further disappointment with the availability of new negative data from large randomized clinical trials. As the first studies were conducted in Asian countries, there may be some doubt about whether efficacy is greater in Asian patients. However, this is unlikely, and Japanese investigators have already criticized the widespread use of favipiravir as a compassionate use as too hasty and poorly justified [33].

In conclusion, favipiravir, administered for ten days, did not improve the assessed outcomes compared with a placebo to treat COVID-19 patients with pneumonia admitted to the hospital. These results align with the results from previous randomized trials. However, in the present study the non-serious adverse events were more frequent in the favipiravir group.
